# Who Needs to Be Allocated in ICU after Thoracic Surgery? An Observational Study

**DOI:** 10.1155/2016/3981506

**Published:** 2016-07-17

**Authors:** Liana Pinheiro, Ilka Lopes Santoro, Sonia Maria Faresin

**Affiliations:** Respiratory Division, Universidade Federal de São Paulo (UNIFESP), 04023-062 São Paulo, SP, Brazil

## Abstract

*Background*. The effective use of ICU care after lung resections has not been completely studied. The aims of this study were to identify predictive factors for effective use of ICU admission after lung resection and to develop a risk composite measure to predict its effective use.* Methods*. 120 adult patients undergoing elective lung resection were enrolled in an observational prospective cohort study. Preoperative evaluation and intraoperative assessment were recorded. In the postoperative period, patients were stratified into two groups according to the effective and ineffective use of ICU. The use of ICU care was considered effective if a patient experienced one or more of the following: maintenance of controlled ventilation or reintubation; acute respiratory failure; hemodynamic instability or shock; and presence of intraoperative or postanesthesia complications.* Results*. Thirty patients met the criteria for effective use of ICU care. Logistic regression analysis identified three independent predictors of effective use of ICU care: surgery for bronchiectasis, pneumonectomy, and age ≥ 57 years. In the absence of any predictors the risk of effective need of ICU care was 6%. Risk increased to 25–30%, 66–71%, and 93% with the presence of one, two, or three predictors, respectively.* Conclusion*. ICU care is not routinely necessary for all patients undergoing lung resection.

## 1. Introduction

The current limitations on financial resources available for healthcare pose a serious challenge worldwide, but particularly in developing countries. At the same time the introduction of advanced health technologies has led to greater family/patient expectations with regard to treatment options and outcomes. These two factors have led to an increased demand for high tech treatment options, including the use of intensive care unit (ICU) services, which add substantial cost to patients' hospitalization.

In Brazil, ICU admission is routine following lung resection. In Brazil and other developing countries, lung resection is done not only for lung cancer, which constitutes the principal indication for this surgical procedure, but also for bronchiectasis or sequel of pulmonary tuberculosis. Many candidates for lung resection are elderly and have comorbidities that could increase the occurrence of life-threatening events, which might contribute to increased demand for ICU care. However, many of these patients are hemodynamically stable and can be extubated immediately after the surgical procedure. Regardless, they are routinely referred to the ICU for surveillance. This indiscriminate use of ICU services following lung resection contradicts the European Respiratory Society/European Society of Thoracic Surgeons guideline recommendation that admission to the ICU after thoracotomy should not be done on a regular basis. Moreover, it might lead to clinical problems such as increased risk of nosocomial infection or delirium and to ICU bed shortage and increase in hospital expenditure [1–3].

Few studies have investigated characteristics of patients undergoing lung resection to identify factors associated with effective use of ICU care in the postoperative period [4–6]. The aims of this study are to identify predictive factors for effective use of ICU admission after lung resection and to develop a risk composite measure to predict effective use.

## 2. Methods

### 2.1. Study Design

We performed an observational prospective cohort study from July 2009 to April 2012. 120 patients over 18 years of age, complete preoperative evaluation, who were undergoing elective lung resection for diagnosed or suspected benign/malignant lung disease who underwent lung resection, were enrolled consecutively. Exclusion criteria were as follows: another surgical procedure in addition to lung resection, no resection of lung parenchyma, and death during intraoperative period.

All patients signed the informed consent for the study before preoperative procedures. This study was approved by the local Ethics Committee of the Universidade Federal de São Paulo (CEP 0410/09; Chairperson: Professor Dr. José Osmar Medina Pestana) on 30 April 2009.

### 2.2. Baseline Assessments

Preoperative evaluation was collected using a structured data collection format. The following data were recorded: complete clinical history, physical examination, age, gender, smoking history, significant comorbidities, routine blood tests, electrocardiography, pulse oximetry, American Society of Anesthesiologists (ASA) score [7–9], pre- and postbronchodilator spirometry, cardiopulmonary exercise tests (CPET), pulmonary perfusion scan, fiber optic bronchoscopy, and thoracic computed tomography scans. Thirty-one study subjects were evaluated for carbon monoxide lung diffusion capacity measurement (DL_CO_). The preoperative physiologic evaluation was performed according to the American College of Chest Physicians (ACCP) Guideline [10] adapted to our institution scenario. Operability criteria were predicted by predicted postoperative forced expiratory volume in one second (ppoFEV_1_) ≥40%, predicted postoperative carbon monoxide lung diffusion capacity (ppoDL_CO_) ≥40%, and maximum oxygen consumption (VO_2_max) at cycloergospirometry ≥ 10 mL/Kg/min.

### 2.3. Intraoperative Assessments

All operations were carried out by the same team of chest surgeons and at the same tertiary referral hospital. The surgical team included certified thoracic surgeons and residents.

After anesthesia induction, a double-lumen tube was inserted. Thoracic epidural anesthesia was initiated intraoperative and continued postoperatively until chest-drain removal or at least 72 hours. Moreover, the protective anesthetic regimen with low tidal volume was not performed in regular basis. The number of patients that were extubated in the operating room was also recorded. Excessive fluid administration during lung resections was avoided in order to minimize pulmonary injury [11].

All patients had lung resections (minor or major) via open thoracotomy (no VATS).

The intraoperative variables recorded were anesthesia time, operative procedure, and surgical and medical complications.

### 2.4. Postoperative Assessments

After surgery, pain control was achieved by intravenous or epidural analgesia. All patients were transferred to the ICU, which was staffed by critical care intensivist physicians who are pulmonologists. The nurse-to-patient ratio was 0.5 and dedicated paramedical personnel (nursing staff and chest physiotherapists) were available.

Postoperative evaluation was also collected using a structured data collection format. Cardiopulmonary and surgical complications, as well as mortality, were recorded. Patients were transferred to the thoracic surgery ward when they were in stable cardiopulmonary condition.

In the postoperative period, the patients were stratified into two groups according to the use of ICU admission: effective use of ICU group and ineffective use of ICU group.

Effective use of ICU was defined as the presence of one or more of the following characteristics postoperatively: maintenance of controlled ventilation after surgery or reintubation; acute respiratory failure; hemodynamic instability, shock or intraoperative/postanesthesia complications including hemodynamic instability, cardiac arrhythmia, blood hypertension, and bronchospasm.

### 2.5. Statistical Methods

Categorical variables were summarized as absolute and relative frequencies (percentage). Chi-squared test or the Fisher exact test was used to compare categorical variables as appropriate. Continuous data were presented as mean and standard deviation (SD), median and interquartile range [IQR]. Student's *t*-test or the Mann-Whitney test was used to compare continuous variables as appropriate.

After the univariate regression analysis, the variables with greater odds ratio, without interactions, were included in a multiple logistic regression analysis, conducted using the stepwise model to identify independent predictive factors for effective use of ICU admission. The evaluation of the adjusted model was based on −2-log likelihood and Nagelkerke *R* ^2^. Age ≥ 57 years (variable median), surgery for bronchiectasis, pneumonectomy, and ASA score were the variables included in the final model. Next, the predictive model coefficient and the possible risk factor combinations were computed. These predictions were then compared with the observed adverse outcome rates by using various discrepancy measurements.

Results were considered significant if the *p* value was less than 0.05. Analysis was performed by the Statistical Package for the Social Sciences for Windows (SPSS) version 19.0.

## 3. Results

The general characteristics of the 120 patients included in this study are summarized in Table 1. The average age was 56.2 years old. The majority were male (54.2%) and current or ex-smoker (62.5%). The means of spirometric parameters were within the normal range. The great majority received an ASA classification of 2 (92.3%) and 65.8% had a diagnosis of malignant disease.

The mean duration of anesthesia was 6.1 ± 1.8 hours. Sixteen pneumonectomies (13.4%), one bilobectomy (0.8%), 58 (48.3%) lobectomies, 16 (13.4%) segmentectomies, two resections of three segments, and 27 (22.5%) other minor procedures were carried out. The greater part of patients (111 patients – 92.5%) was extubated in the operating room.

A total of 39 patients (32.5%) had 89 complications. Eighteen patients (15%) experienced clinical complications, nine (7.5%) surgical, and 12 (10%) both. Five patients (4.2%) developed bronchopleural fistula, 15 patients (12.5%) had pneumonia, seven patients (5.8%) had arrhythmia, and none patient experienced myocardial infarct.

The 30-day mortality rate was 2.5% or three patients. The cause of death was septic shock, a result of pulmonary infection in one patient and bronchopleural fistula in two patients. These patients had been diagnosed with bronchiectasis and* Aspergillus* coinfection. Two also had COPD.

The characteristics of patients included in the effective use and ineffective use of ICU groups are shown in Table 2. A significantly higher proportion of patients in the effective use group had respiratory symptoms (93%), comorbidities, ASA classification of 3 (17%), pneumonectomies (27%), and benign diseases (50%) compared to the ineffective use group. Mean anesthesia time was significantly longer for the effective use group compared to ineffective use group (6.9 h versus 5.9 h); no difference was found between the groups in terms of gender, age, smoking history, or spirometry values.

On the first postoperative day, 12 patients (40%) from the effective use of ICU group were clinically stable and were transferred to the thoracic surgery ward. All patients in the ineffective use group were transferred to the thoracic surgery ward on the first postoperative day.

The morbidity rate in the effective use of ICU group was 100% and the mortality was 6.6%, while in the ineffective use group the morbidity and mortality were 10% and 1.1%, respectively.

Logistic regression analysis showed that, after adjusting for ASA score of 3 (reference < 3), the surgical treatment of bronchiectasis, pneumonectomy, and age ≥ 57 years old were independent predictors for the effective use of ICU admission in the postoperative period of lung resection (odds ratio 6.7, 5.8, and 5.2, resp.) (Table 3).

The regression coefficients were used in an equation to predict the risk of effective use of ICU in the postoperative period of pulmonary resection considering the presence or absence of three risk factors: surgical treatment of bronchiectasis, pneumonectomy, and age ≥ 57 years. From this equation we obtained eight risk groups of effective use of ICU which could be clustered into four groups: no predictor, 6% risk; one risk predictor, 25 to 30% risk; two predictors, 66 to 71% risk; three predictors, 93% risk (Table 4). Taking as reference the first group (no predictor), the risk for effective use of ICU in the postoperative period of lung resection in the presence of one, two or three predictors increased the risk of effective use of ICU by 5, 10, and 15 times, respectively.

## 4. Discussion

This observational prospective cohort study evaluated the effective use of admission to the ICU after lung resection among patients in Sao Paulo, Brazil. Patients in the effective use of ICU group were clinically worse than those in the ineffective use group. More patients in the effective use group had comorbidities and respiratory symptoms. A higher proportion of patients in the effective use group were classified as ASA 3. In addition there were more pneumonectomies, longer anesthesia time, and greater number of benign diseases, particularly bronchiectasis, in the effective use group.

Logistic regression analysis confirmed that surgical treatment of bronchiectasis, pneumonectomy, and age ≥ 57 years was independent predictors for the effective use of ICU. The final model established eight incremental risk groups, which could be reduced to four risk groups. The risk of effective use of ICU after lung resection was roughly equal to the baseline risk (6%) times the number of independent predictors (one, two, or three) multiplied by 5.

Two important aspects should be taken into account in terms of minimizing the ICU admission; firstly the literature has shown some evidence concerning pulmonary rehabilitation in the preoperative scenario for patients undergoing lung resection surgery [12]. In our study, the preoperative rehabilitation was not feasible due to the short time interval between referral and surgery or logistic reasons. Secondly, it is essential to highlight that the protective anesthetic regimen (low tidal volume and low driving insufflation pressure) was applied specially during pneumonectomy procedure.

In our study all patients were referred to the ICU because there is no intermediate care unit at our institution able to provide adequate monitoring and intensive nursing care. Among the 30 patients who required ICU by our defined criteria, 12 (40%) were clinically compensated and were transferred to the thoracic surgery ward on the first postoperative day. These patients might have been admitted to an intermediate care unit had one been available.

Only three previous studies have investigated predictors of ICU use in the postoperative period of lung resection [4–6]. None were done in developing countries. Two of three studies included only patients with lung cancer and major resections. The present study included major and minor resections in patients with malignant and benign diseases. This decision was based on the fact that, in developing countries such as Brazil, thoracic surgery for bronchiectasis and for posttuberculosis pulmonary sequel is commonplace.

Despite the fact that benign lung diseases like bronchiectasis generally affect younger patients, the surgical morbidity and mortality rates are higher for benign disease than for malignant disease. Studies over the last decade showed morbidity rates from 18 to 53% and mortality from zero to 26.3% for benign disease [13–17]. In the present study, the morbidity and mortality rates among patients with bronchiectasis were 56% and 12%, respectively, confirming our preliminary clinical observation and justifying the inclusion of this group of patients in our sample.

Although the characteristics of the study population in the present study were different from those of the previous studies on effective use of ICU care following lung resection, some of the conclusions from this study are similar to those of the previous studies. In 2006, Pieretti et al. [4] published a retrospective study to evaluate factors predicting the use for intensive care in patients undergoing major lung resection. Twenty eight per cent of the study subjects effectively required intensive care. Similar to the present study, this group of patients was older and had higher rates of comorbidities, higher ASA scores, and higher number of pneumonectomies. This group had also lower PaO_2_, FEV_1_, ppoFEV_1_, DL_CO_, ppoDL_CO_, and PPP (product of % ppoFEV_1_ and ppoDL_CO_). Stepwise regression generated two models. The first identified PPP and ASA score as independent predictors of effective use of ICU care; the second identified ppoDL_CO_ and ASA score as independent predictors.

Brunelli et al. [5], in 2008, developed and validated the first scoring system to predict ICU admission for complications after major lung resections. Emergency admission to the ICU was considered when the patient developed major cardiopulmonary complications requiring advanced life-support treatments. Among the 1297 patients included in that study 6.3% had ICU admission for complications and 1.6% died. Pneumonectomy, age > 65 years, ppoFEV_1_ below 65%, ppoDL_CO_ below 50%, and cardiac comorbidity were identified as independent predictors of ICU use. The ICU scoring system was developed by proportional weighting of the logistic regression coefficients and was validated by the authors, as well as by a second group, Okiror et al. [6], who found the scoring system to have a moderate discriminating ability to predict the risk for ICU admission after lung resection.

In our study, a higher number of pneumonectomies were observed in the effective use of ICU group and pneumonectomy was identified as an independent predictor of effective ICU use. Brunelli et al. [5] also identified pneumonectomy as an independent predictor. In that study, when pneumonectomy was the only risk factor, the risk of requiring ICU care ranged from 10 to 20%, while in the present study the risk was 27%. This relatively low risk suggests that patients with only pneumonectomy as a risk factor may be candidates for an intermediate care unit, rather than an ICU. This could save substantial costs as many studies have shown that hospital costs rises geometrically when ICU is the local of the treatment [18].

Brunelli et al. [5] found also that age over 65 years was a predictive factor of use for ICU after lung resection. Age was also found to be predictive in the present study, but the value, ≥ 57 years, was lower.

ASA classification was identified as a predictive factor for ICU use following lung resection in the present study and in the study by Pieretti et al. [4] ASA classification is a widespread tool that is easy to use [8, 19]. Although it is a good predictor of morbidity and mortality [20] it is vital to reinforce that the ASA score was not designed to identify individual anesthetic or surgical risk [9]. Moreover, the ASA scale may lack scientific precision, since some studies have demonstrated a lack of reliability among anesthesiologists in assigning ASA scores [7, 8]. Another important point of controversy is inconsistent application of the term “systemic disease.” Bronchiectasis with repeated or significant hemoptysis is not considered “systemic diseases” in the ASA classification and therefore is not considered in the classification [19]. In our study, that disease strongly affected the risk of effective use of ICU admission.

The three previous studies identified DL_CO_ as an independent predictive factor for ICU use following lung resection. We were not able to fully evaluate DL_CO_ as a potential predictor because of limited test availability at our institution. The DL_CO_ was only performed on 31 selected patients in our study group, according to the American College of Chest Physicians (ACCP) Guideline [10] adapted to our institutional scenario.

The main limitation of our study is the sample size which was restricted by the fact that patients were recruited from a single centre. However, there were several advantages to the single centre design: operative procedures were performed by the same surgical team, preoperative clinical evaluation was performed by the same clinical team, and the postoperative and ICU care was homogenous.

In conclusion, although all of the patients in this study were admitted to the ICU following lung resection, only 25% exhibited effective use for ICU care. Three independent predictors were identified for effective use: surgery for bronchiectasis, pneumonectomy, and age ≥ 57 years. Without any of these predictors, the risk of requiring ICU care was 6%. The presence of one, two, or three predictors increased the risk of effective use of ICU care by 5, 10, and 15 times, respectively. These results suggest that ICU care is not routinely necessary for all patients undergoing lung resection. Even patients with one risk factor may not require ICU care if an intermediate care unit is available. Hospitals should consider the costs and benefits of establishing intermediate care units.

## Figures and Tables

**Table 1:** Characteristics of patients included in the study.

Variables	Value
Patient, *n*	120
Male, *n* (%)	65 (54.2)
Age and years, mean (SD)	56.2 (12.3)
Respiratory symptoms, median [IQR]	2 [0–3]
Comorbities, median [IQR]	2 [1–3]
Charlson index, median [IQR]	3 [2–4]
Current or ex-smoker, *n* (%)	75 (62.5)
ASA physical status, *n* (%)	
1	4 (3.3)
2	111 (92.5)
3	5 (4.2)
Functional parameters, % predict (*n*), mean (SD)	
FVC (120)	88.4 (17.6)
FEV_1_ (120)	82.3 (19.2)
FEV_1_/FVC (120)	0.75 (0.1)
ppoFEV_1_ (120)	72.4 (19.3)
DL_CO_ (31)	64.4 (19.0)
ppoDL_CO_ (31)	53.4 (15.2)
Diagnosis after surgery, *n *(%)	
Benign	41 (34.2)
Malignant	79 (65.8)
Primary lung cancer	52 (65.8)
Metastatic lung cancer	27 (34.2)

IQR: interquartile ratio; DL_CO_: carbon monoxide lung diffusion capacity; FEV_1_: forced expiratory volume in one second; FVC: forced volume capacity; *n*: number of patients; ppoDL_CO_: predicted postoperative carbon monoxide lung diffusion capacity; ppoFEV_1_: predicted postoperative forced expiratory volume in one second; SD: standard deviation.

**Table 2:** Comparison of effective use of ICU group ineffective use of ICU group characteristics.

	Effective use of ICU		*p*
	Yes *n* = 30	No *n* = 90	
Male, *n* (%)	17 (57%)	48 (53%)	0.75
Age and years, mean (SD)	58 (13)	55 (12)	0.23
Smokers and ex-smokers, *n* (%)	19 (63%)	56 (62%)	0.91
Respiratory symptoms, *n* (%)	28 (93%)	60 (67%)	0.004
Comorbidities, median [IQR]	2 [2-3]	2 [1–3]	0.04
Charlson index, median [IQR]	3 [2–4]	3 [2–4]	0.26
ASA			
1	0 (0%)	4 (5%)	<0.001
2	25 (83%)	86 (95%)	
3	5 (17%)	0 (0%)	
Functional tests, % predict, mean SD			
FVC	85 ± 19	89 ± 17	0.22
FEV_1_/FVC	0.73 ± 0.14	0.75 ± 0.10	0.38
ppoFEV_1_	66 ± 17	74 ± 20	0.06
DL_CO_	60 ± 20 (*n* = 31)	67 ± 18 (*n* = 31)	0.38
ppoDL_CO_	51 ± 16 (*n* = 31)	55 ± 15 (*n* = 31)	0.51
Anaesthesia time, hours, mean (SD)	6.9 (1.9)	5.9 (1.7)	0.008
*n* resected segments, median [IQR]	3 [1.75–9]	3 [0–5]	0.18
Pneumonectomy, *n* (%)	8 (27%)	8 (9%)	0.03
Histopathological diagnosis			
Benign	15 (50%)	26 (29%)	0.025
Malign	13 (43%)	39 (43%)	
Metastasis	2 (7%)	25 (28%)	

ASA: American Society of Anaesthesiologists; DL_CO_: carbon monoxide lung diffusion capacity; FEV_1_: forced expiratory volume in one second; FVC: forced volume capacity; IQR = interquartile ratio; *n*: number; ppoDL_CO_: predicted postoperative carbon monoxide lung diffusion capacity; ppoFEV_1_: predicted postoperative forced expiratory volume in one second; SD: standard deviation.

**Table 3:** Multivariate analysis of the three most important risk factors of effective use of ICU care after lung resection.

Variable	Coefficient	SE	*p*	OR	95% CI
Bronchiectasis	1.905	0.619	0.002	6.72	1.99–22.63
Pneumonectomy	1.757	0.753	0.020	5.79	1.32–25.37
Age ≥ 57 years	1.644	0.604	0.007	5.18	1.58–16.91
Constant	−2.745	0.571	<0.001		

CI: confidence interval; OR: odds ratio; SE: standard error.

**Table 4:** Risk of effective use of ICU after lung resection considering the presence or absence of identified risk factors.

Groups	Bronchiectasis	Pneumonectomy	Age ≥ 57 years	% risk ICU admission
1	No	No	No	6
2	No	No	Yes	25
3	No	Yes	No	27
4	Yes	No	No	30
5	No	Yes	Yes	66
6	Yes	No	Yes	69
7	Yes	Yes	No	71
8	Yes	Yes	Yes	93
